# Ectopic Dpp signaling promotes stem cell competition through EGFR signaling in the *Drosophila* testis

**DOI:** 10.1038/s41598-019-42630-y

**Published:** 2019-04-16

**Authors:** Yanfen Lu, Yuncong Yao, Zhouhua Li

**Affiliations:** 1 College of Plant Science and Technology, Beijing University of Agriculture, No. 7 Beinong Road, Changping District, Beijing, 102206 China; 2 College of Life Sciences, Capital Normal University, Beijing, 100048 China

## Abstract

Stem cell competition could select the fittest stem cells and potentially control tumorigenesis. However, little is known about the underlying molecular mechanisms. Here, we find that ectopic Decapentaplegic (Dpp) signal activation by expressing a constitutively active form of Thickveins (Tkv^CA^) in cyst stem cells (CySCs) leads to competition between CySCs and germline stem cells (GSCs) for niche occupancy and GSC loss. GSCs are displaced from the niche and undergo differentiation. Interestingly, we find that induction of Tkv^CA^ results in elevated expression of *vein*, which further activates Epidermal Growth Factor Receptor (EGFR) signaling in CySCs to promote their proliferation and compete GSCs out of the niche. Our findings elucidate the important role of Dpp signaling in regulating stem cell competition and tumorigenesis, which could be shed light on tumorigenesis and cancer treatment in mammals.

## Introduction

Tissue homeostasis is maintained by adult stem cells, which constantly divide and supply newly differentiated cells to replace dying or damaged cells. Increasing evidence shows that fittest stem cells are constantly selected through stem cell competition, which is critical for organ development and tissue homeostasis^1–6^. Moreover, stem cell competition is found to be implicated in tumorigenesis^7,8^. However, the underlying molecular mechanisms of stem cell competition are poorly understood.

The *Drosophila* testis is an ideal system to study stem cell maintenance, differentiation, and competition^9–38^. A group of non-dividing somatic cells, termed the hub, resides at the apex of the *Drosophila* testis^14,22,26^. About 5–9 GSCs closely attach to the hub via adhesion molecules. Another group of somatic stem cells, termed CySCs, attach to the hub by their cellular extensions^10,12,13,26^. The hub serves as the stem cell niche and expresses Unpaired (Upd), which activates the Janus Kinase/Signal Transducer and Activator of Transcription (JAK/STAT) signaling in GSCs and CySCs to control their maintenance^3,32,39^. GSCs undergo asymmetric divisions, producing new GSCs and differentiating gonialblasts (GBs). The GBs are engulfed by two somatic cyst cells, generated from asymmetric CySC divisions. The GBs undergo four rounds of mitotic division with incomplete cytokinesis before differentiation. The somatic cyst cells grow without further division to encapsulate the germline cells with their cellular extensions throughout spermatogenesis^12,13,17,26,27,40,41^. CySCs are also critical for GSC maintenance, therefore, CySCs together with the hub define the niche for GSCs^3,32,33,42^.

Bone Morphogenetic Protein (BMP) and Hedgehog (Hh) signaling play important roles in the maintenance of GSCs and CySCs^20,21,23,24,29,30,43–46^. The hub and the early cyst cells produce two BMP ligands, Glass bottom boat (Gbb) and Dpp^43,44^. Short-range BMP signaling is critical for GSC maintenance and differentiation. BMP production and diffusion within the niche must be tightly controlled to ensure localized BMP signaling inside the niche, while ectopic BMP signaling outside of the niche leads to aberrant GSC proliferation and differentiation^45,47–52^. Our recent study found that Tkv functions as ligand sink to spatially restrict Dpp signaling within the testis niche^53^. However, it remains unknown whether ectopic Dpp signaling in CySCs has any role in stem cell regulation.

CySCs and GSCs often compete for niche occupancy, making the *Drosophila* testis an excellent model to study the underlying mechanisms controlling stem cell competition. Stem cell competition selects fittest stem cells for tissue homeostasis, and is potentially implicated in tumorigenesis^1–5^. Previous studies found that CySCs compete with each other and with GSCs for niche occupancy. The mutant stem cell and its descendants with increased competitiveness will outcompete wild type stem cells^4,6,15,16,19,24,46,54^. In the *Drosophila* testis, CySC-GSC competition is first revealed in *socs36E* mutant, the negative regulator of JAK/STAT signaling^16^. Recent studies found that several signaling pathways, including Hh, Hippo (Hpo), and EGFR/Mitogen-activated protein kinase (MAPK), regulate stem cell competition^15,19,24,46,54^. However, the underlying mechanisms controlling stem cell competition are not fully understood.

In this study, we investigate whether additional factors regulate stem cell competition in the testis niche. Interestingly, we find that ectopic expression of *tkv*^*CA*^ in CySCs results in competition between CySCs and GSCs for niche occupancy and GSC loss. We demonstrate that CySC-GSC competition observed in *tkv*^*CA*^-expressing testis is caused by enhanced expression of the EGF *vein* (*vn*), which in turn activates EGFR/MAPK signaling in CySCs to promote CySCs to outcompete GSCs. Our data elucidate a novel mechanism of stem cell competition, which may shed light into the development of potential clinical treatment for cancer.

## Results

### Ectopic expression of *tkv*^*CA*^ in CySCs leads to CySC-GSC competition and GSC loss

**Figure 1:**
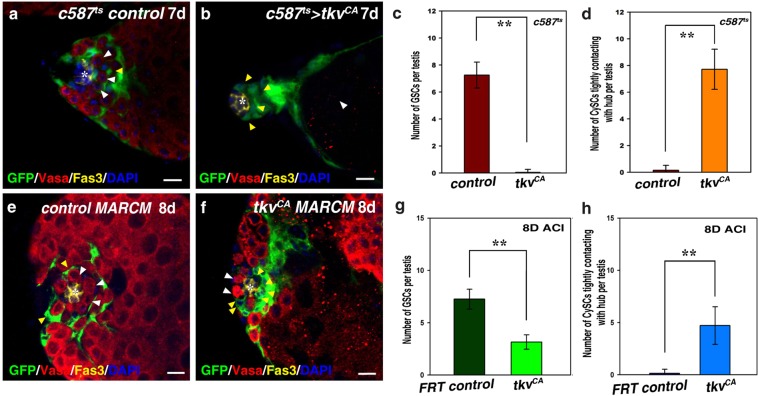
Ectopic expression of *tkv*^*CA*^ in CySCs leads to GSC loss. (**a**) *c587*^*ts*^ control testis. GSCs (white arrowheads) and CySCs (yellow arrowhead) are indicated. (**b**) *c587*^*ts*^ > *tkv*^*CA*^ testis. The hub is surrounded by CySCs (yellow arrowheads), and no germline cells can be observed (white arrowhead). (**c**) Quantification of the number of GSCs per testis in control and *c587*^*ts*^ > *tkv*^*CA*^ testes. *n* = 10–15 testes. (**d**) Quantification of the number of CySCs with their cell body attaching to the hub in control and *c587*^*ts*^ > *tkv*^*CA*^ testes. *n* = 10–15 testes. (**e**) CySC MARCM clones in *FRT* control. GSCs (white arrowheads) and GFP-marked CySCs (yellow arrowhead) are indicated. (**f**) *tkv*^*CA*^-expressing CySC MARCM clones. Some GFP-marked *tkv*^*CA*^ CySCs (yellow arrowheads) tightly attach to the hub, and the number of GSCs per testis is greatly reduced (white arrowhead). (**g**) Quantification of the number of GSCs per testis in testes carrying *FRT* control and *tkv*^*CA*^ MARCM clones. *n* = 10 testes. (**h**) Quantification of the number of CySCs with their cell body attaching to the hub in testes carrying *FRT* control and *tkv*^*CA*^ MARCM clones. *n* = 10 testes. mean ± SEM is shown. **p < 0.01. GFP in green, Vasa in red, Fas3 in yellow, blue indicates DAPI staining for DNA. Scale bars: 10 μm.

In order to search for new regulators of stem cell competition, we performed a large-scale screen using a *c587*^*ts*^ driver (*c587Gal4*, *UAS-GFP; esg-lacZ*, *tubGal80*^*ts*^) (data not shown)^53^. *c587Gal4* is strongly expressed in CySCs and somatic cyst cells of the *Drosophila* testis (Fig. 1a). Our recent data show that Tkv acts as receptor trap to restrain Dpp signaling within the niche^53^. Surprisingly, we found that when a constitutively active form of *tkv* (*tkv*^*CA*^) was expressed in CySCs (*c587*^*ts*^ > *tkv*^*CA*^), all germline cells, including GSCs, were lost (Fig. 1b). The hub was tightly surrounded by a group of somatic cells, instead of GSCs (Fig. 1b–d). These data indicate that ectopic expression of *tkv*^*CA*^ may cause CySC-GSC competition. The observed phenotype was resulted from systemic expression of *tkv*^*CA*^ in all CySCs, we wondered whether ectopic expression of *tkv*^*CA*^ in single CySC or only a portion of CySCs could cause the same defect. We explored this possibility by using MARCM technique to generate CySC clones expressing *tkv*^*CA*^. Compared with *FRT* control CySC clones, we found that *tkv*^*CA*^*-*expressing CySC clones tightly attached to the hub, and the number of GSCs per testis was significantly decreased (Fig. 1e–h). These data indicate that ectopic *tkv*^*CA*^ expression in CySCs causes stem cell competition.

Mad (Mothers against dpp), a transducer of Dpp signaling, is phosphorylated when the Dpp pathway is activated. Therefore, the accumulation of phosphorylated Mad (pMad) can be used as a read-out of Dpp pathway activation^43,55,56^. Consistent with previous reports that ectopic expression of *tkv*^*CA*^ induces ectopic Dpp signaling activation^43,55^, we found that Dpp signaling activation was greatly increased in the cyst cell lineage of *c587*^*ts*^ > *tkv*^*CA*^ testis, using pMAD as a readout (Supplementary Fig. 1). As Dpp signaling is highly activated upon ectopic expression of *tkv*^*CA*^ in CySCs (Supplementary Fig. 1), we examined whether the observed stem cell competition phenotype was a consequence of ectopic Dpp signaling. We used various functional RNAi lines to simultaneously deplete components downstream of Tkv in *c587*^*ts*^ > *tkv*^*CA*^ testes^53,57^. Mad and Med (Medea) are components downstream of Tkv in the Dpp signaling pathway. When these RNAi constructs were co-expressed with *tkv*^*CA*^ in CySCs, we found that further removal of either Mad or Med could successfully suppress stem cell competition observed in *c587*^*ts*^ > *tkv*^*CA*^ testes (Supplementary Fig. 2). In these testes, GSCs were restored and resided around the hub, and differentiating spermatogonia could be observed (Supplementary Fig. 2). These data demonstrate that stem cell competition and GSC loss resulted from ectopic expression of *tkv*^*CA*^ in CySCs is a consequence of ectopic Dpp signaling.

### CySCs overproliferate and outcompete GSCs upon *tkv*^*CA*^ expression

**Figure 2:**
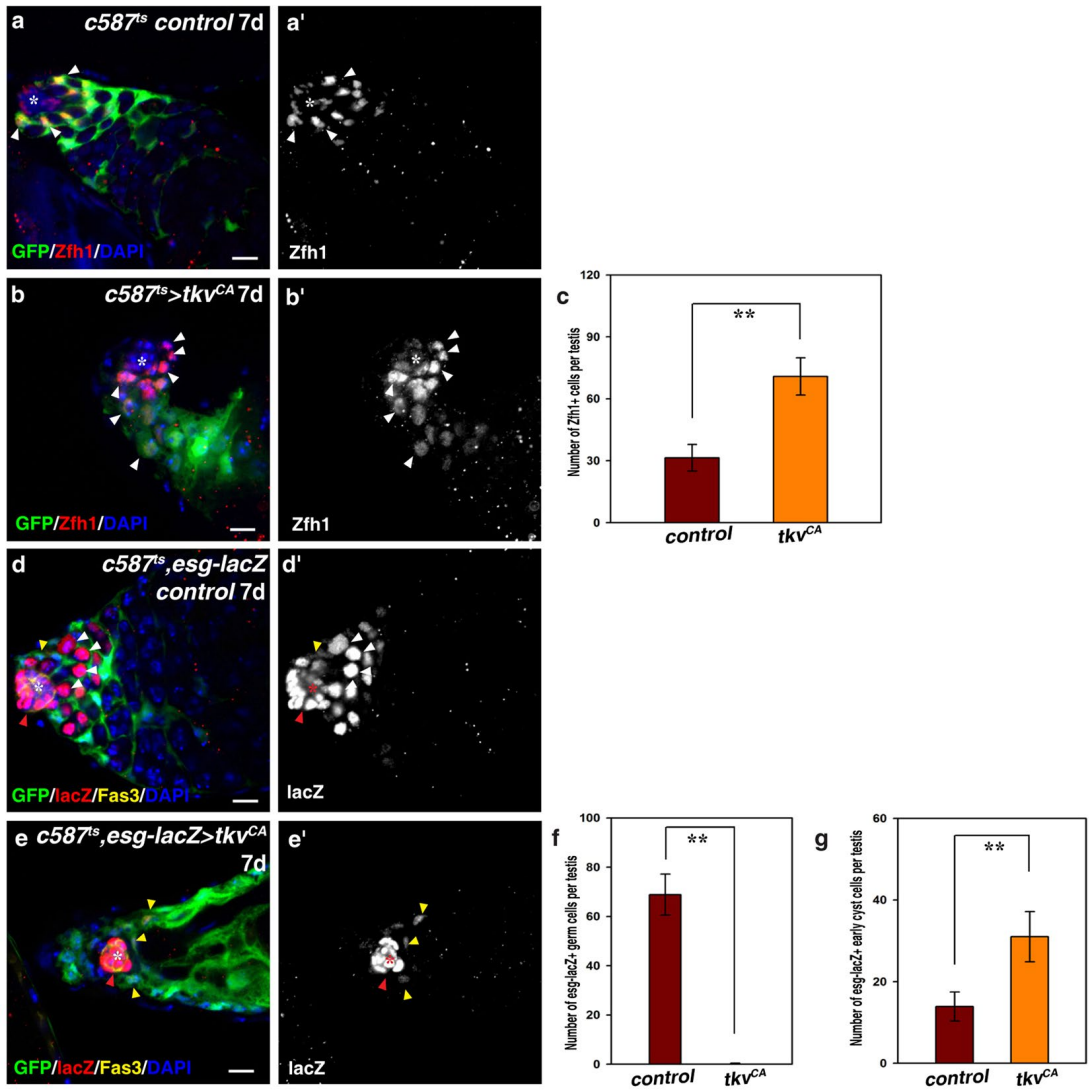
Ectopic expression of *tkv*^*CA*^ in CySCs leads to CySC overproliferation and CySC-GSC competition. (**a**) CySCs (by Zfh1, red, white arrowheads) in *c587*^*ts*^ control testis. (**b)** CySCs (white arrowheads) in *c587*^*ts*^ > *tkv*^*CA*^ testis. (**c**) Quantification of the number of Zfh1^+^ cells in control and *c587*^*ts*^ > *tkv*^*CA*^ testes. *n* = 10–15 testes. (**d**) *esg-lacZ* (by lacZ, red) in *c587*^*ts*^ control testis. *esg-lacZ* is highly expressed in the hub (red arrowhead and asterisk) and the early germline cells (GSCs and GBs) (white arrowheads), and weakly expressed in CySCs (yellow arrowhead). (**e**) *esg-lacZ* (red) in *c587*^*ts*^ > *tkv*^*CA*^ testis. No germline cells can be observed. *esg-lacZ* can only be observed in the hub (red arrowhead and asterisk) and CySCs (yellow arrowheads). (**f**) Quantification of the number of *esg-lacZ*^+^ germline cells in control and *c587*^*ts*^ > *tkv*^*CA*^ testes. *n* = 10–15 testes. (**g**) Quantification of the number of *esg-lacZ*^+^ early cyst cells in control and *c587*^*ts*^ > *tkv*^*CA*^ testes. *n* = 10–15 testes. mean ± SEM is shown. **p < 0.01. GFP in green, blue indicates DAPI staining for DNA. Scale bars: 10 μm.

Next, we investigated the cell identity of the cells in *c587*^*ts*^ > *tkv*^*CA*^ testes. We first examined *c587*^*ts*^ > *tkv*^*CA*^ testes using the Zfh1 antibody, which labels CySCs and early cyst cells. The number of Zfh1^+^ cells was significantly increased compared to control testes, and CySCs tightly attached to the hub with their cell bodies, indicating that ectopic Dpp signaling in CySCs promotes CySC proliferation (Fig. 2a–c; data not shown). We then examined these *c587*^*ts*^ > *tkv*^*CA*^ testes using *esg-lacZ*, which was highly expressed in the hub and GSCs, and at low levels in CySCs (Fig. 2d). Interestingly, no germline cells were observed in these testes (Fig. 2e,f). *esg-lacZ* could only be observed in the hub and CySCs, and the number of *esg-lacZ*^+^ cells was dramatically increased (Fig. 2e,g). These data show that upon ectopic expression of *tkv*^*CA*^, CySCs continued to proliferate and occupied the whole niche, while the germline cells were completely lost.

### GSCs are competed out of the niche and undergo differentiation upon *tkv*^*CA*^ expression

**Figure 3:**
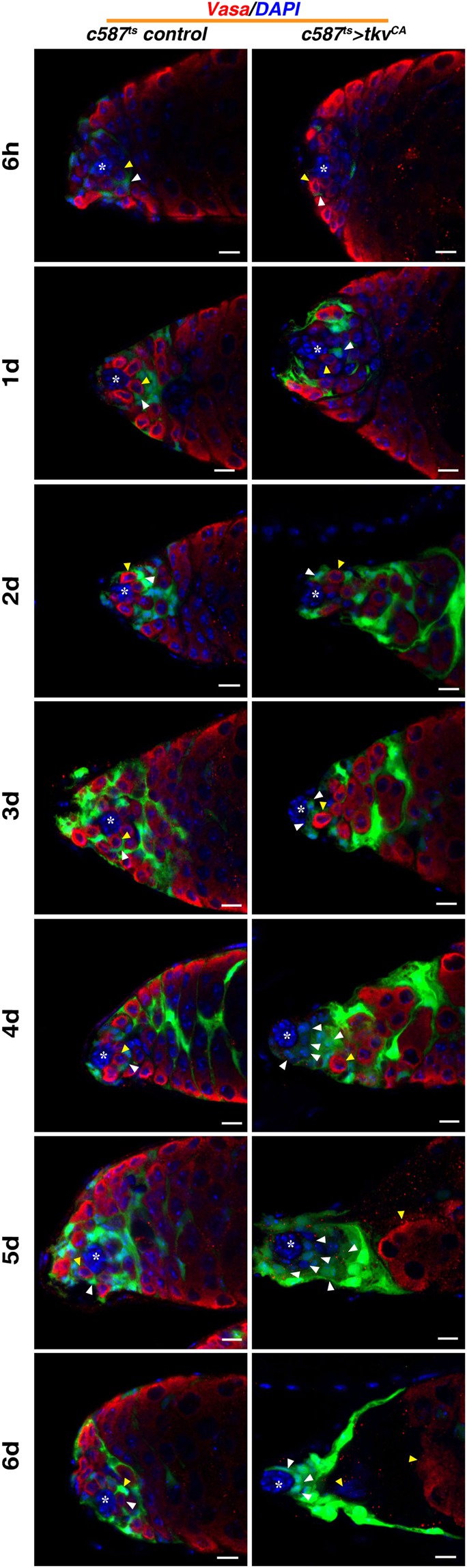
GSCs are competed out of the niche by CySCs and differentiated in *c587*^*ts*^ > *tkv*^*CA*^ testis. Time chase experiment is carried out to trace the fate of GSCs. Time points examined are indicated. The hub is marked by white asterisk, GSCs are indicated by yellow arrowheads, and CySCs by white arrowheads. CySCs begin to closely attach to the hub on the 2^nd^ day at 29 °C from 18 °C. The hub is closely associated by CySCs on the 3^rd^ day after shifting, and all GSCs are competed out of the niche by CySCs and undergo differentiation (white arrowhead). Germline cells move further away from the hub and undergo differentiation by the 6^th^ day. Almost all the spermatogonia are terminally differentiated, and fully differentiated spermatids can be observed (yellow arrowhead). GFP in green, blue indicates DAPI staining for DNA. Scale bars: 10 μm.

As no germline cells were observed in these testes, we examined the fate of the germline cells, especially GSCs. The complete disappearance of germline cells, especially GSCs, may be caused by differentiation or cell death. To distinguish these two possibilities, we first performed time chase experiments. No differences were observed between the control and *c587*^*ts*^ > *tkv*^*CA*^ testes at 6 hours and 24 hours after the flies were shifted from 18 °C to 29 °C (Fig. 3; Supplementary Fig. 3). However, by the 2nd day, we found that some CySCs closely attached to the hub with their cell bodies in the *c587*^*ts*^ > *tkv*^*CA*^ flies, and the number of GSCs per testis was decreased (Fig. 3; Supplementary Fig. 3). By the 3rd day after shifting, we found that the hub was closely associated by CySCs and all GSCs were competed out of the niche (Fig. 3; Supplementary Fig. 3). As time lapsed, GSCs were pushed further away from the hub by CySCs and underwent differentiation. By the 6th day, almost all germline cells were terminally differentiated, and fully differentiated spermatids could be observed at regions near the hub (Fig. 3; Supplementary Fig. 3). CySCs closely attached to the hub kept proliferating, resulting in accumulation of CySCs (Fig. 3; Supplementary Fig. 3). On the contrary, we did not find any significant increase of GSC/germline cell death (by active Caspase-3) in these testes (Supplementary Fig. 4). These data indicate that ectopic activation of Dpp signaling in CySCs outcompetes GSCs from the niche by CySCs, and the outcompeted GSCs are lost due to differentiation.

### Ectopic Dpp signaling in CySCs promotes Vn expression

Previous report found that increased expression of the adhesion protein integrin in *socs36E* mutant CySCs could promote CySC-GSC competition^16^. We examined whether CySC-GSC competition observed in *c587*^*ts*^ > *tkv*^*CA*^ testes was due to elevated expression of integrin. However, no obvious change in integrin levels was observed (by βPS-integrin), indicating that CySC-GSC competition observed in *c587*^*ts*^ > *tkv*^*CA*^ testes is unlikely mediated by integrin molecules (Supplementary Fig. 5).

**Figure 4:**
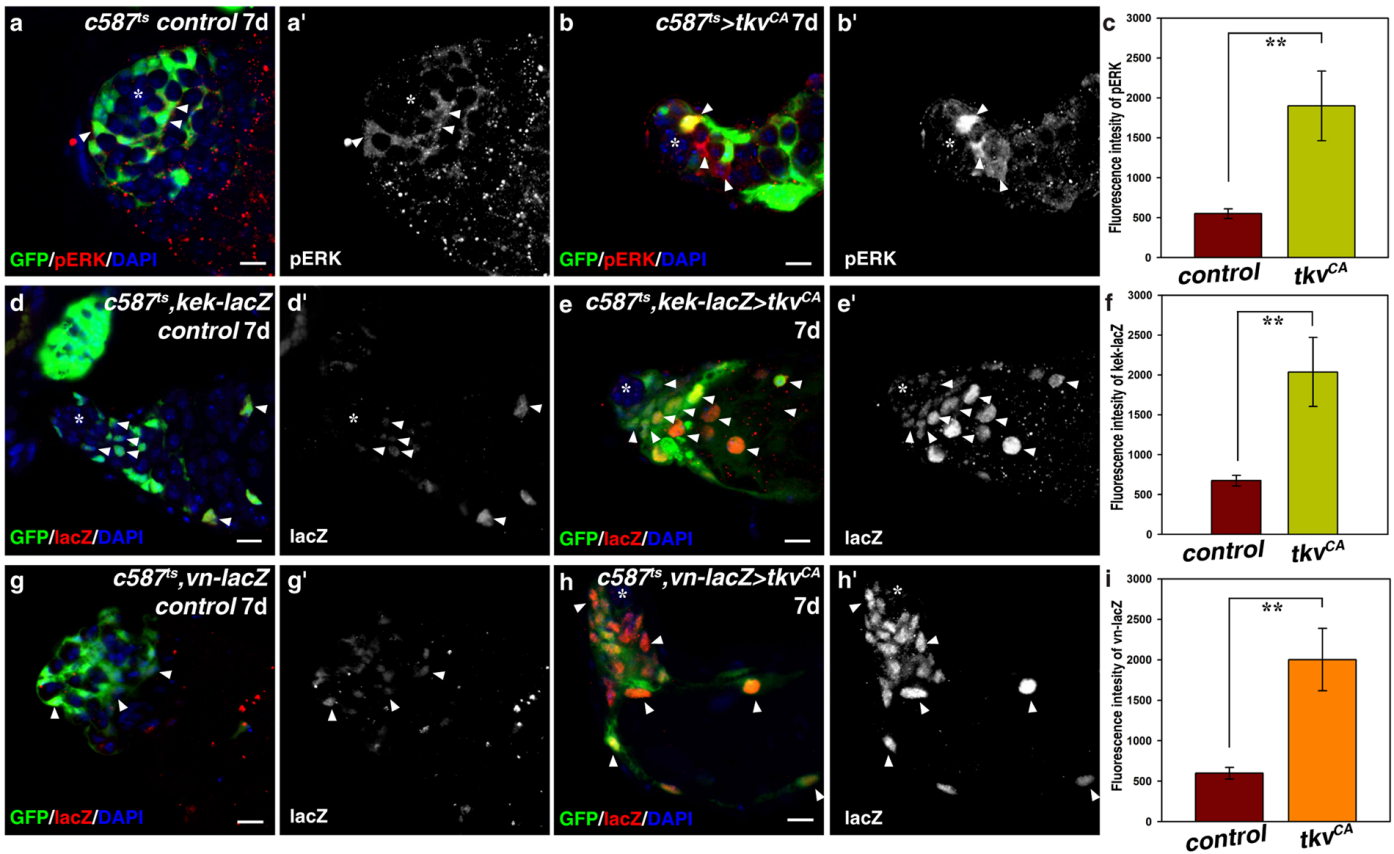
Ectopic Dpp signaling in CySCs promotes *vn* expression. (**a**) EGFR signaling activation (by pERK, red) in *c587*^*ts*^control testis. pERK signal is mainly observed in the early cyst cells (white arrowheads). (**b**) EGFR signaling is highly activated in the somatic cyst cells of *c587*^*ts*^ > *tkv*^*CA*^ testis. (**c**) Quantification of fluorescence intensity of pERK in control and *c587*^*ts*^ > *tkv*^*CA*^ testes. *n* = 10. (**d**) EGFR signaling activation (by *kek-lacZ*, red) in *c587*^*ts*^ control testis. *kek-lacZ* is expressed in the somatic cyst cells (white arrowheads). (**e**) *kek-lacZ* is highly expressed in *c587*^*ts*^ > *tkv*^*CA*^ testis. (**f**) Quantification of the fluorescence intensity of *kek-lacZ* in control and *c587*^*ts*^ > *tkv*^*CA*^ testes. *n* = 10. (**g**) *vn* expression (by *vn-lacZ*, red) in *c587*^*ts*^ control testis. *vn-lacZ* is expressed at low levels in the early cyst cells (white arrowheads). (**h**) *vn-lacZ* is highly expressed in *c587*^*ts*^ > *tkv*^*CA*^ testis. (**i**) Quantification of the fluorescence intensity of *vn-lacZ* in control and *c587*^*ts*^ > *tkv*^*CA*^ testes. *n* = 10. mean ± SEM is shown. **p < 0.01. GFP in green, blue indicates DAPI staining for DNA. Scale bars: 10 μm.

Previous studies found that elevated EGFR signaling in *socs36E*- and *Madm*-deficient CySCs was responsible for CySC-GSC competition^15,19^. Activation of EGFR by its extracellular ligands triggers a signal transduction cascade, mediated by the Ras/Raf/MEK cassette, which ultimately leads to dual phosphorylation and activation of the mitogen-activated protein kinase/extracellularly regulated kinase (MAPK/ERK), therefore, phosphorylated ERK (pERK) can be used as a read-out of EGFR pathway activation^58^. To investigate whether EGFR signaling is responsible for CySC-GSC competition observed in *c587*^*ts*^ > *tkv*^*CA*^ testes, we examined the activation of EGFR signaling by detecting the levels of pERK in *tkv*^*CA*^-expressing CySCs. Interestingly, we found the levels of pERK was significantly increased in *tkv*^*CA*^-expressing CySCs than those in the control, indicating that ectopic Dpp signaling promotes the activation of EGFR signaling (Fig. 4a–c). To further confirm this, we examined the expression of *kekkon* (*kek*), a primary downstream target of EGFR signaling. *kek-lacZ* is an enhancer trap that reflects endogenous *kek* expression^59^. We found that *kek-lacZ* was expressed in the early cyst cells and differentiated cyst cells in wild type testis (Fig. 4d). The expression pattern of *kek-lacZ* is similar to that of pERK, indicating that *kek-lacZ* could be used as a readout of EGFR activation in testis (Fig. 4a,d)^60^. We found *tkv*^*CA*^ induction significantly enhanced the expression levels of *kek-lacZ* (Fig. 4e,f). These data show that ectopic Dpp signaling significantly promotes EGFR signaling in the somatic cyst cells.

We then explored how EGFR signaling was activated by ectopic *tkv*^*CA*^ expression. We reasoned that some components of the EGFR signaling pathway may be transcriptional upregulated by the activated MAD/MED complex, which in turn activate EGFR signaling. We thus investigated whether ectopic *tkv*^*CA*^ expression promotes the transcription of EGFs. We examined the expression of EGFs (Spitz (Spi) and Vein (Vn)) in the testis using their enhancer traps. Consistently, we found that *spi* was expressed in the germline cells (by *spi-lacZ*) (Supplementary Fig. 6)^61^. While *vn* was expressed in the early somatic cyst cells, including CySCs (by *vn-lacZ*) (Fig. 4g)^17,19^. As *tkv*^*CA*^ was ectopically expressed in CySCs, therefore, we focused on *vn* for further examination. To explore the relationship between ectopic Dpp signaling and *vn* expression, we examined the expression levels of *vn* in *c587*^*ts*^ > *tkv*^*CA*^ testes. We found *vn* expression was markedly increased upon *tkv*^*CA*^ induction (Fig. 4h,i). These data suggest that ectopic Dpp signaling promotes *vn* expression, which in turn induces elevated EGFR signaling in the early cyst cells.

### Ectopic Vn/EGFR/MAPK signaling is responsible for CySC-GSC competition

**Figure 5:**
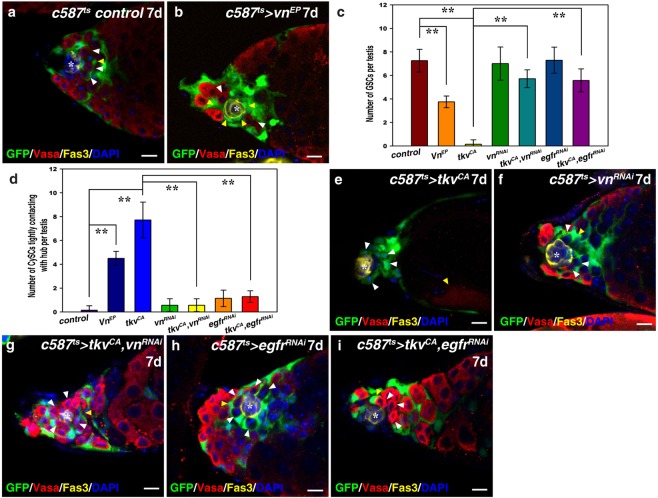
Ectopic Vn/EGFR/MAPK signaling is responsible for CySC-GSC competition and GSC loss in *c587*^*ts*^ > *tkv*^*CA*^ testes. (**a**) *c587*^*ts*^ control testis. (**b**) *c587*^*ts*^ > *vn*^*EP*^ testis. Note that most GSCs (red, white arrowheads) are competed out of the niche by CySCs (yellow arrowheads). (**c**) Quantification of the number of GSCs per testis in the testis with indicated genotype. *n* = 10–15 testes. (**d**) Quantification of the number of CySCs with their cell body attaching to the hub per testis with indicated genotypes. *n* = 10–15 testes. (**e**) *c587*^*ts*^ > *tkv*^*CA*^ testes. (**f**) *c587*^*ts*^ > *vn*^*RNAi*^ testes (HMS00004). No obvious defects are observed when this shRNA is induced. (**g**) Stem cell competition observed in *c587*^*ts*^ > *tkv*^*CA*^ testis is almost completely suppressed by simultaneous expression of this shRNA against *vn*. (**h**) *c587*^*ts*^ > *egfr*^*RNAi*^ testes. The induction of this weak *egfr*^*RNAi*^ line (JF01368) in CySCs does not cause any obvious defects. (**i**) Stem cell competition observed in *c587*^*ts*^ > *tkv*^*CA*^ testis is greatly suppressed by co-expression of this *egfr*^*RNAi*^. mean ± SEM is shown. **p < 0.01. GFP in green, Vasa in red, Fas3 in yellow, blue indicates DAPI staining for DNA. Scale bars: 10 μm.

Therefore, we addressed whether elevated *vn* expression was responsible for CySC-GSC competition observed in *c587*^*ts*^ > *tkv*^*CA*^ testes. When ectopically expressed in CySCs (*c587*^*ts*^ > *vn*^*EP*^), we found that *vn*^*EP*^ overexpression resulted in CySC-GSC competition, which mimics *c587*^*ts*^ > *tkv*^*CA*^ testes (Fig. 5a–d). Consistently, the number of GSCs per testis was significantly reduced in *c587*^*ts*^ > *vn*^*EP*^ testes (Fig. 5a–c), and the number of CySCs tightly attaching to the hub was greatly increased in *c587*^*ts*^ > *vn*^*EP*^ testes compared with that of control testes (Fig. 5b,d). These data indicate that elevated *vn* expression promotes CySC-GSC competition. Furthermore, we found that expression of a constitutively active form of Ras (Ras^V12^) also resulted in CySC-GSC competition and GSC loss, phenocopying *tkv*^*CA*^ expression (data not shown). These data indicate that CySC-GSC competition observed in *c587*^*ts*^ > *tkv*^*CA*^ testes is likely a consequence of ectopic EGFR/MAPK signaling.

To further confirm that elevated Vn/EGFR/MAPK signaling is responsible for CySC-GSC competition observed in *c587*^*ts*^ > *tkv*^*CA*^ testes, we performed suppression experiments. No obvious defects was caused when *vn* was depleted in CySCs using a shRNA (TH03149.N) (Fig. 5f). When *vn* was compromised in *c587*^*ts*^ > *tkv*^*CA*^ testes using this shRNA, the observed CySC-GSC competition and GSC loss defects were almost completely suppressed (Fig. 5e–g). The number of GSCs per testis and the number of CySCs tightly attaching to the hub were almost completely reverted by simultaneous knockdown of *vn* (Fig. 5a–d). These results indicate that ectopic *vn* expression is responsible for CySC-GSC competition observed in *c587*^*ts*^ > *tkv*^*CA*^ testes. To further confirm our conclusion, we targeted EGFR itself for suppression assay. As EGFR signaling is essential for spermatogonia differentiation, we selected a weak dsRNA against *egfr* (JF01368) to inhibit EGFR signaling^17,37,62^. Knockdown of *egfr* using this dsRNA resulted in no obvious defects (Fig. 5h). We found that the observed CySC-GSC competition and GSC loss defects were almost completely suppressed by simultaneous induction of this dsRNA in *c587*^*ts*^ > *tkv*^*CA*^ testes (Fig. 5e,h,i). Consistently, the number of GSCs per testis and the number of CySCs tightly attaching to the hub were almost completely suppressed by co-inhibition of *egfr* (Fig. 5c,d). Together, these data demonstrate that ectopic Vn/EGFR/MAPK signaling is responsible for CySC-GSC competition and GSC loss resulted from *tkv*^*CA*^ expression in CySCs.

## Discussion

Fittest stem cells are selected through stem cell competition in the niche to maintain tissue homeostasis. However, the mechanisms underlying stem cell competition remain largely unknown. Here, we reveal that cell-autonomous activation of Dpp signaling in CySCs results in CySC-GSC competition and GSC loss, which is mediated by elevated Vn/EGFR/MAPK signaling. The mechanism we uncovered may be general features of stem cell systems in regulating stem cell competition^2,22,63^.

Stem cell competition emerges as a mechanism to select fit stem cells and control tumorigenesis^1–5^. Stem cell competition takes place in three steps. The competitive stem cells first become more fit, before they move and anchor to a defined niche, followed by proliferation and outcompetition of neighboring stem cells. However, the detailed mechanisms underlying stem cell competition in the *Drosophila* testis are poorly understood. Elucidating the mechanisms controlling stem cell competition will help to develop potential clinic treatments for cancer. The testis niche supports two groups of stem cells: GSCs and CySCs, making it an excellent model to study stem cell competition regulation. Previous studies found that CySCs compete with each other and with GSCs for niche occupancy^15,16,19,46^. Mutations that confer increased competitiveness to CySCs result in outcompetition of wild type resident stem cells by the mutant stem cells and their descendants. The first identified regulator of niche competition is Socs36E, a negative feedback inhibitor of the JAK/STAT pathway. The competitive behavior of *socs36E* mutant CySCs was first attributed to increased JAK/STAT signaling^16^. However, it was recently found that the competitiveness of *socs36E* mutant CySCs is likely due to elevated MAPK signaling^15^. Stem cell competition also occurs among CySCs, it was reported that CySCs with increased Hh or Yorkie (Yki) activity displaced neighboring wildtype CySCs from the niche before they outcompeted neighboring wild type GSCs, indicating that both intra- (CySC-CySC) and inter-lineage (CySC-GSC) competitions take place in the testis^46^. It was recently reported that Slit-Robo signaling only regulates intra-lineage competition among CySCs^54^.

Ectopic Dpp signaling in CySCs results in CySC-GSC competition for niche anchoring and GSC loss (Fig. 1). We found that ectopic Dpp signaling leads to elevated Vn expression, which in turn activates EGFR/MAPK signaling in CySCs to promote their proliferation and ability to outcompete GSCs for niche occupancy (Figs 4 and 5). Ectopic expression of *vn* in CySCs results in CySC-GSC competition, which mimics *c587*^*ts*^ > *tkv*^*CA*^. However, the GSC loss and the CySC overproliferation phenotype in *c587*^*ts*^ > *vn*^*EP*^ is not as severe as the latter. The differences may be caused by the *vn*^*EP*^ line used in this study, which may not produce sufficient *vn* transcripts as that of *tkv*^*CA*^ expression. Nevertheless, the observed CySC-GSC competition upon *tkv*^*CA*^ expression is almost completely suppressed by compromising EGFR signaling (Fig. 5). Our study here demonstrate that the niche signals must be tightly controlled to prevent CySC-GSC competition, thereby maintaining niche homeostasis. Interestingly, a recent study found that the novel tumor suppressor Mlf1-adaptor molecule (Madm) regulates CySC-GSC competition^19^. They found that Madm regulates CySC-GSC competition by suppressing the expression of integrin and EGFR ligand Vn^19^. Although *tkv*^*CA*^ induction promotes *vn* expression, we found that, unlike loss of *madm*, *tkv*^*CA*^ induction does not affect integrin expression levels, suggesting that the downstream events regulating stem cell competition in *tkv*^*CA*^ and *madm*^−/−^ CySCs are not identical (Supplementary Fig. 5). It is established that EGFR/MAPK signaling is required in the somatic cyst cells for their proper differentiation and engulfment of the developing germline cells^17,37,62^. From recent studies on *socs36E*, *Madm*, and our study on *tkv*^*CA*^, we can conclude that EGFR/MAPK signaling in CySCs also plays a pivotal role in regulating CySC-GSC competition^15,19^. It will be interesting to investigate why BMP signaling is kept from being over-activated in CySCs under physiological conditions, and how different input signals are converged on the EGFR/MAPK signaling pathway to regulate CySC-GSC competition, which will help to understand the regulation of stem cell competition, tissue homeostasis, and tumorigenesis.

## Materials and Methods

### Fly lines and cultures

Flies were maintained on standard corn-meal cultural media at 25 °C. To inactivate Gal80^ts^, flies were shifted to 29 °C, and transferred to new vials every day and dissected at specific time points as indicated. Information about alleles and transgenes used can be found in FlyBase and as noted: *c587Gal4*, *UAS-GFP*, *esg-lacZ*, *tubGal80*^*ts*^ (*c587*^*ts*^), *UAS-tkv*^*Q253D*^ (*tkv*^*CA*^), *UAS-mad*^*RNAi*^ (GL01527, GLV21013, JF01263, JF01264, NIG 12399R-1, and 12399R-2), *UAS-med*^*RNAi*^ (JF02218 and GL01313), *kek*^*BB142*^ (*kek-lacZ*, gift from Zhaohui Wang), *spi*^*s3547*^ (*spi-lacZ*, gift from Rongwen Xi), *vn*^*p1749*^ (*vn-lacZ*, gift from Rongwen Xi), *UAS-vn*^*RNAi*^ (TH03149.N, Tsinghua University), *UAS-egfr*^*RNAi*^ (JF01368), *vn*^*EPg35521*^ (BL58498), *UAS*-*w*^*RNAi*^ (BL33613 and HMS00004) (from TRiP at Harvard Medical School)^64^.

### RNAi knock down and overexpression experiments

To examine gene function in CySCs, *c587*^*ts*^ (*c587Gal4*, *UAS-GFP*, *esg-lacZ*, *tubGal80*^*ts*^) was used. Crosses were maintained at 18 °C. Progeny with the proper genotypes was collected 1–2 days after eclosion and maintained at 29 °C before examination. *UAS-dsRNA* and *UAS-shRNA* transgenic flies were used.

### MARCM clone analyses

CySC MARCM clones were generated by heat shock treatment^65^. 1–3 days old adult flies were heat-shocked at 37 °C for 60 minutes for 2 consecutive days. Flies were maintained at 25 °C and transferred to new vials every day. The clones were assayed at indicated time points after clone induction (ACI).

### Immunostainings and fluorescence microscopy

For fluorescent immunostainings, testes were dissected in 1 × PBS, and fixed in 4% paraformaldehyde for 25 min at room temperature. Testes were washed with 1 × PBT (0.1% Triton X-100 in 1 × PBS) for 3 times, 5 min each, and blocked with 3% BSA for 45 min. The samples were incubated with primary antibodies overnight at 4 °C. The following antibodies were used: mouse mAb anti-Fas3 (7G10, 1:50, developed by Corey S. Goodman, Developmental Studies Hybridoma Bank (DSHB)), mouse mAb anti-βPS-integrin (CF.6G11, 1:50, developed by D. Brower, DSHB), rabbit anti-Vasa (d-260, Santa Cruz, 1:200), rabbit anti-Zfh1 (1:5000, a generous gift from Ruth Lehmann, and 1:8000, generated in our lab)^53^, rabbit anti-β-galactosidase (1:5000, Cappel), mouse anti-β-galactosidase (1:1000, Cell Signaling), rabbit anti-pMAD3 (1:300, Epitomics), rabbit anti-active Caspase-3 (1;200, Abcam), and rabbit anti-pERK (p-p44/42, 1:200, Cell Signaling). Primary antibodies were detected by fluorescent-conjugated secondary antibodies (Jackson ImmunoResearch Laboratories). Secondary antibodies were incubated at room temperature for 2 hrs. After secondary antibody staining, DAPI (0.1 μg/ml, Sigma-Aldrich) was added to the samples for 45 min at room temperature. Mounting medium (2.5% DABCO in 70% glycerol) was added to the samples. All images were captured under a Zeiss inverted confocal microscope (780) and were further processed using Adobe Photoshop and Illustrator.

## Supplementary information

SUPPLEMENTARY MATERIAL

## Data Availability

The number of GSCs and CySCs was counted manually. For fluorescence intensity of pERK and *lacZ*, all images were taken under the same confocal settings. Image Pro Plus 5.0 software was used to measure fluorescence intensity of pERK and *lacZ* (using the measure/count function). Statistical analysis was performed using the Student’s *t*-test. PEMS 3.1 software was used for SEM analyses. The graphs were generated using SigmaPlot 10.0 software, and further modified using Adobe Photoshop and Illustrator.
