# Supplementary material for: Ectopic Dpp signaling promotes stem cell competition through EGFR signaling in the Drosophila testis
Source: Sci Rep. 2019 Apr 16;9:6118. doi: 10.1038/s41598-019-42630-y (PMC6467874; doi:10.1038/s41598-019-42630-y)
Supplement: Supplementary file 1 — SUPPLEMENTARY MATERIAL [file 41598_2019_42630_MOESM1_ESM.docx]

**SUPPLEMENTAL MATERIAL**

**Ectopic Dpp signaling promotes stem cell competition through EGFR signaling in the *Drosophila* testis**

Yanfen Lu, Yuncong Yao, and Zhouhua Li


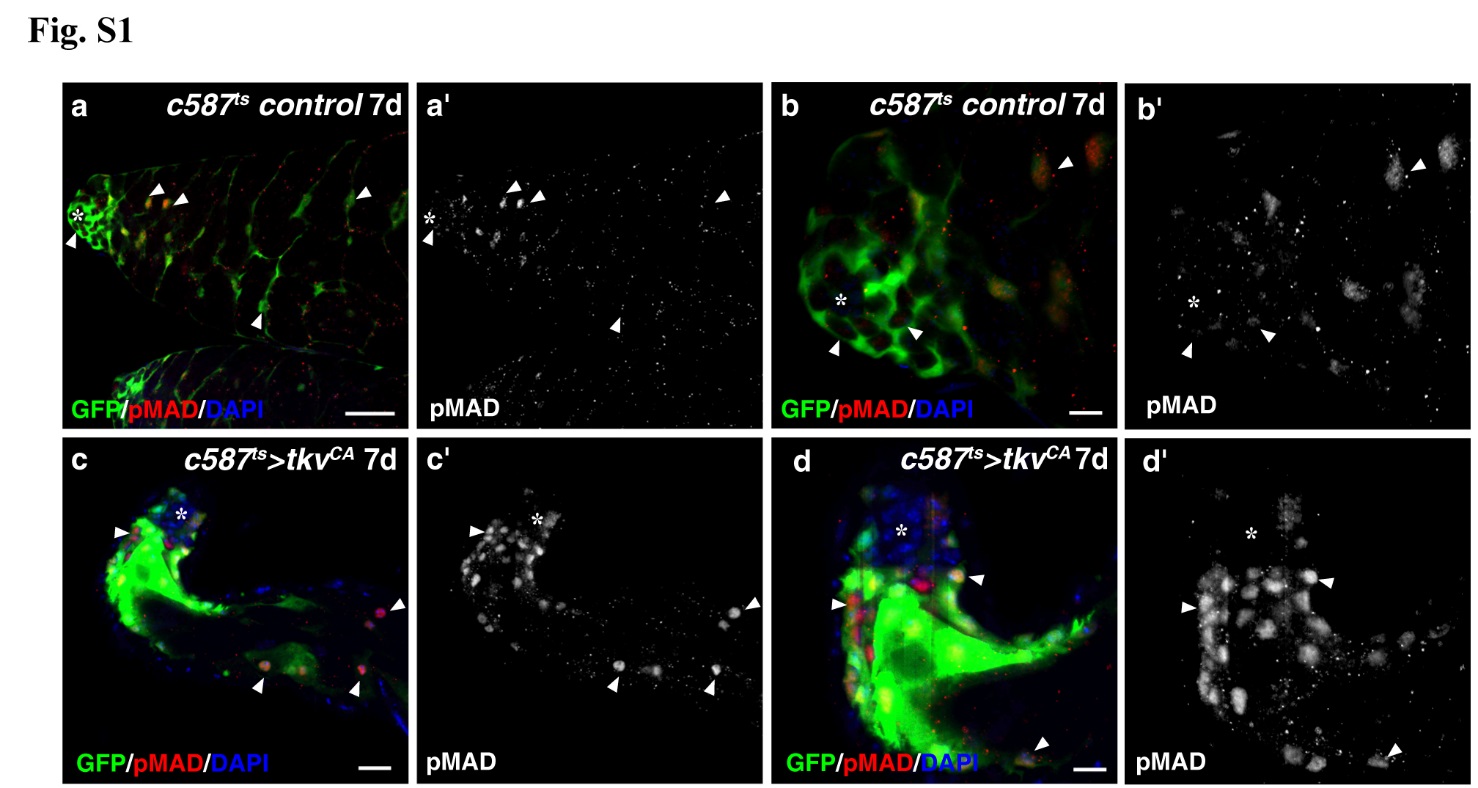


**Supplementary Figure 1.** Dpp signaling is highly activated in the somatic cyst lineage cells of *c587^ts^>tkv^CA^* testis. **a-b'** Dpp signaling activation (by pMAD, red) in *c587^ts^* control testis. Note that Dpp signaling is activated at low levels in GSCs, and higher levels in differentiated cyst cells (white arrowheads). **c**-**d'** Dpp signaling is highly activated (by pMAD, red) in *c587^ts^>tkv^CA^* testis. Note that pMAD signal is highly elevated in *tkv^CA^*-expressing cells (white arrowheads). The hub is marked by white asterisk, pMAD channel in black-white (**a'**-**d'**). GFP in green, blue indicates DAPI staining for DNA. Scale bars: 50 μm (**a** and **c**) and 10 μm (**b** and **d**).


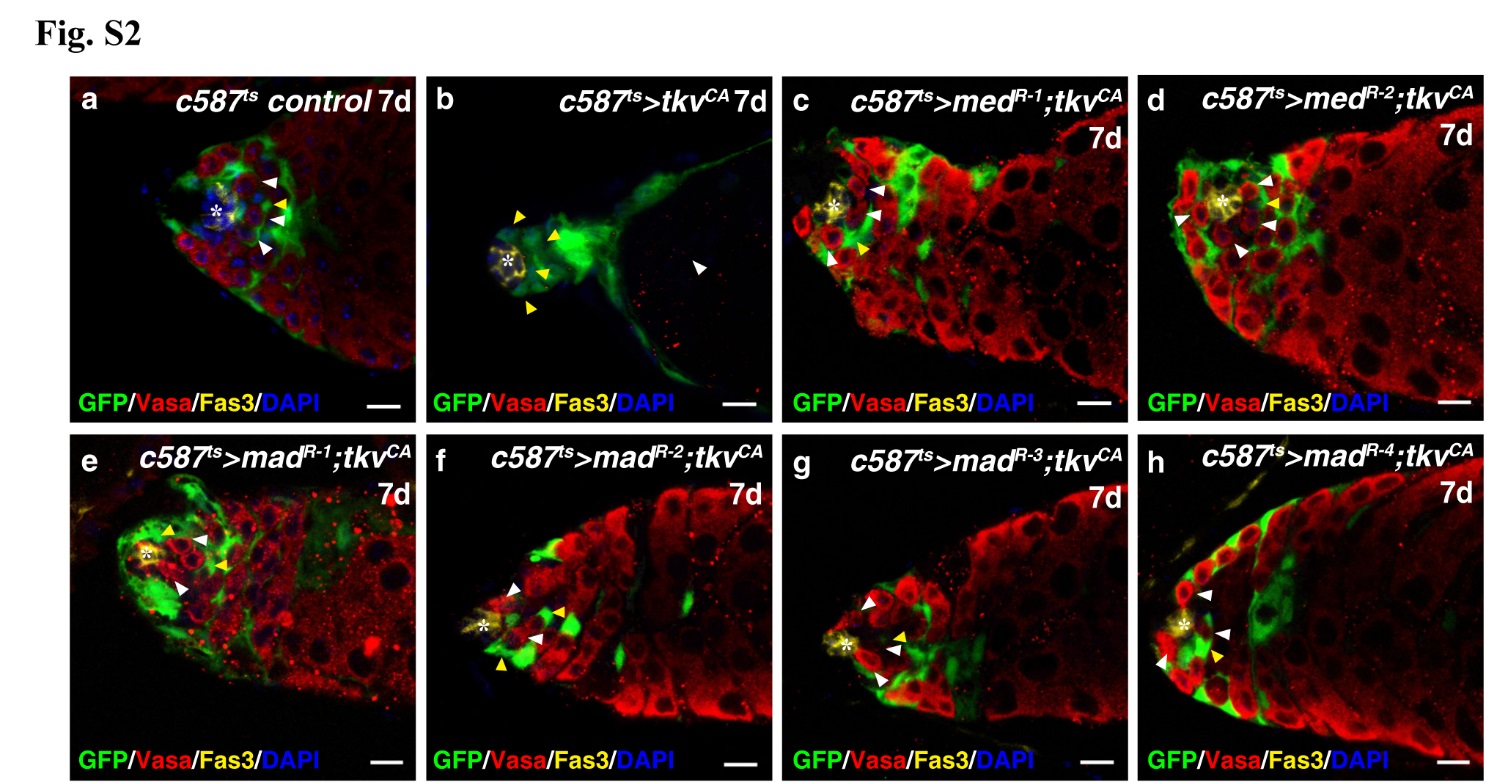


**Supplementary Figure 2.** Stem cell competition observed in *c587^ts^>tkv^CA^* testis is caused by ectopic Dpp signaling. **a** *c587^ts^* control testis. GSCs (white arrowheads) and CySCs (yellow arrowhead) attach to the hub. **b** *c587^ts^>tkv^CA^* testis. The hub is surrounded by CySCs (yellow arrowheads), and no germline cells can be observed (white arrowhead). **c** *c587^ts^>med^RNAi-1^, tkv^CA^* testis. JF02218 is referred as *med^RNAi-1^*. **d** *c587^ts^>med^RNAi-2^, tkv^CA^* testis. GL01313 is referred as *med^RNAi-2^*. **e** *c587^ts^>mad^RNAi-1^, tkv^CA^* testis. GL01527 is referred as *mad^RNAi-1^*. **f** *c587^ts^>mad^RNAi-2^, tkv^CA^* testis. GLV21013 is referred as *mad^RNAi-2^*. **g** *c587^ts^>mad^RNAi-3^, tkv^CA^* testis. JF01263 is referred as *mad^RNAi-3^*. **h** *c587^ts^>mad^RNAi-4^, tkv^CA^* testis. JF01264 is referred as *mad^RNAi-4^*. Note that knockdown of either Med or Mad, the components downstream of Tkv, by different RNAi lines almost completely suppresses stem cell competition caused by *tkv^CA^* expression. The hub is marked by white asterisk, Vasa in red, Fas3 in yellow, GFP in green, blue indicates DAPI staining for DNA. Scale bars: 10 μm.


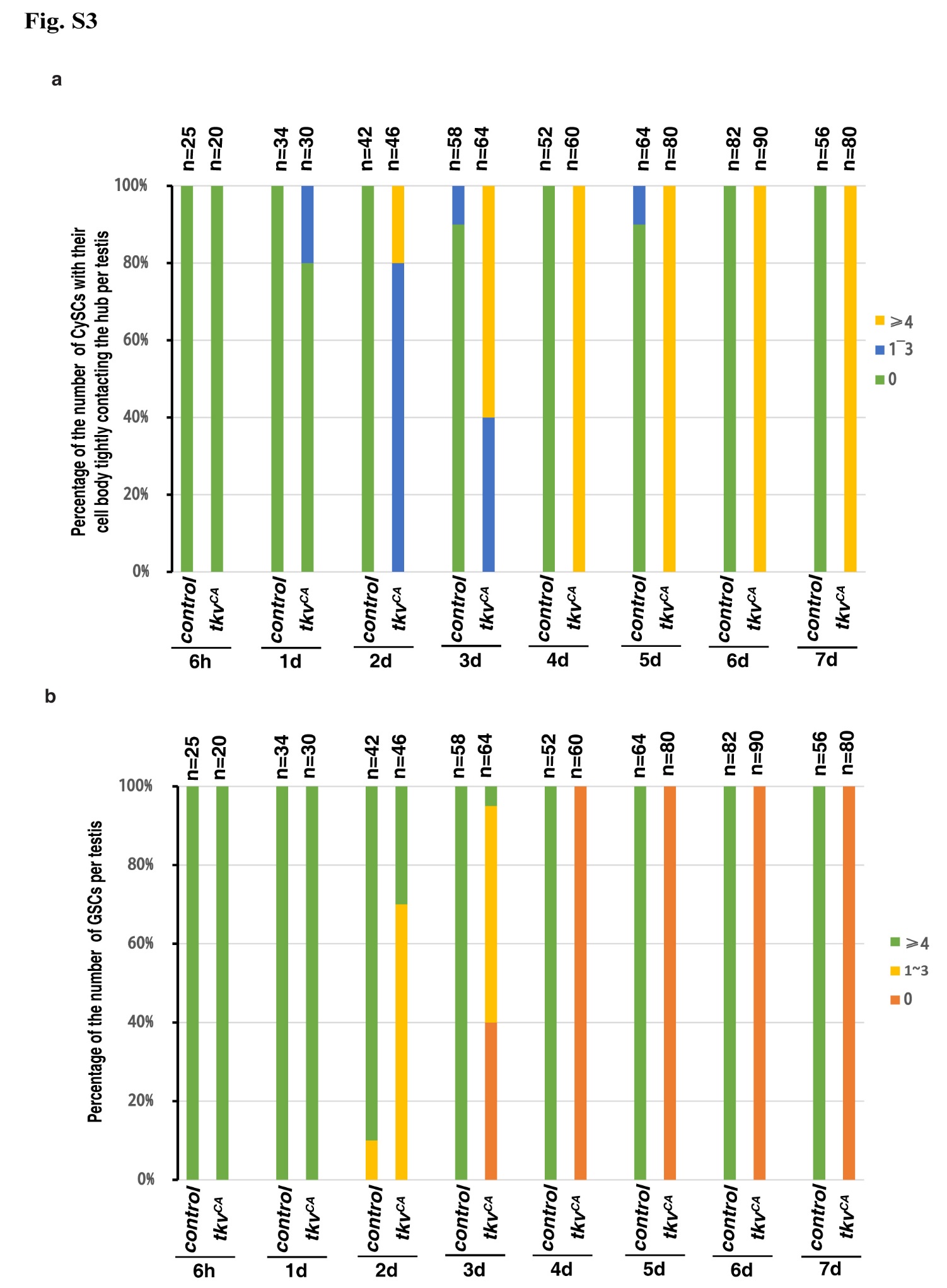


**Supplementary Figure 3.** GSCs are competed out of the niche by CySCs and differentiated in *c587^ts^>tkv^CA^* testis. **a** Quantification of the percentage of the number of CySCs with their cell body attaching to the hub per testis in control and *c587^ts^>tkv^CA^* testes at different time points. **b** Quantification of the percentage of the number of GSCs per testis in control and *c587^ts^> tkv^CA^* testes at different time points. The number of testes examined is indicated. In each testis, the numbers of CySCs with their cell body attaching to the hub and GSCs were counted respectively.


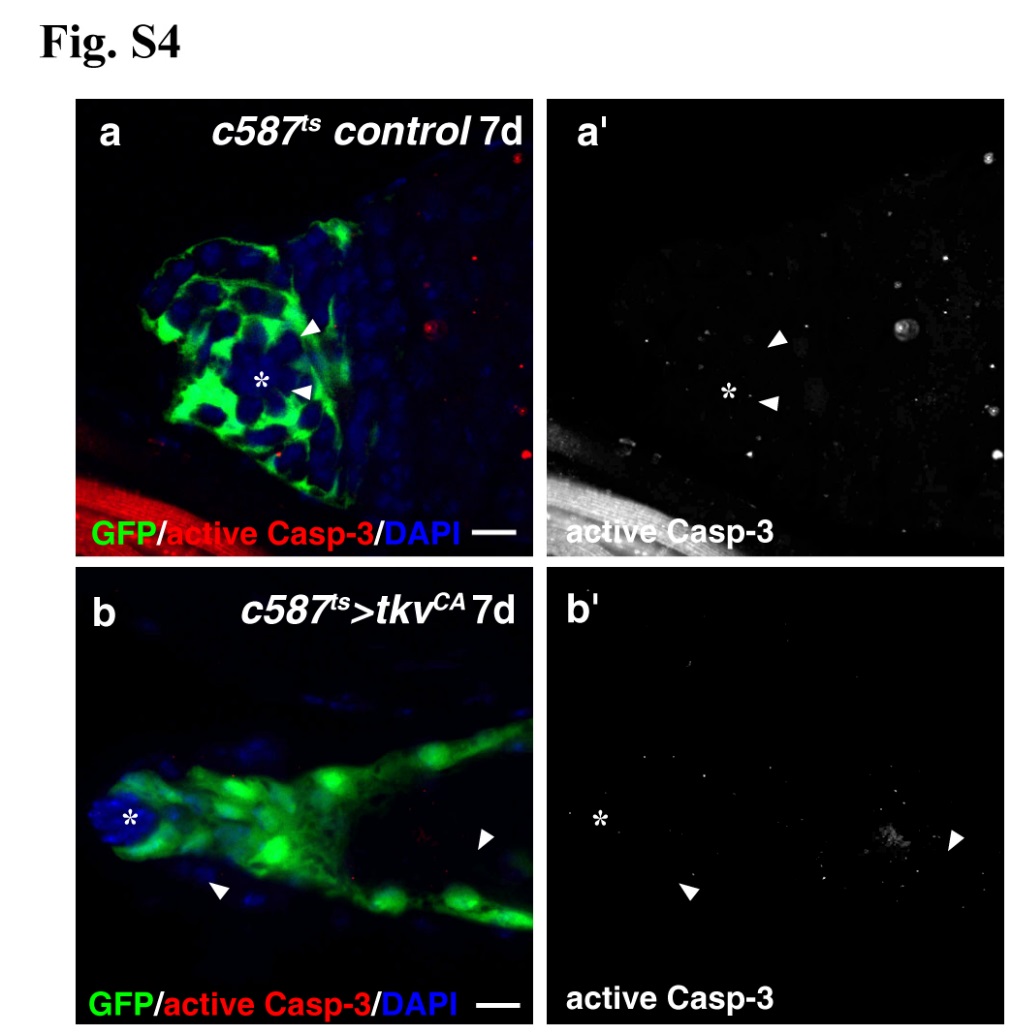


**Supplementary Figure 4.** No increase of active Caspase-3 is observed in *c587^ts^>tkv^CA^* testis. **a** Active Caspase-3 staining (red) in *c587^ts^* control testis (white arrowheads). **b** Active Caspase-3 staining (red) in *c587^ts^>tkv^CA^* testis (white arrowheads). The hub is marked by white asterisk, GFP in green, blue indicates DAPI staining for DNA. Scale bars: 10 μm.


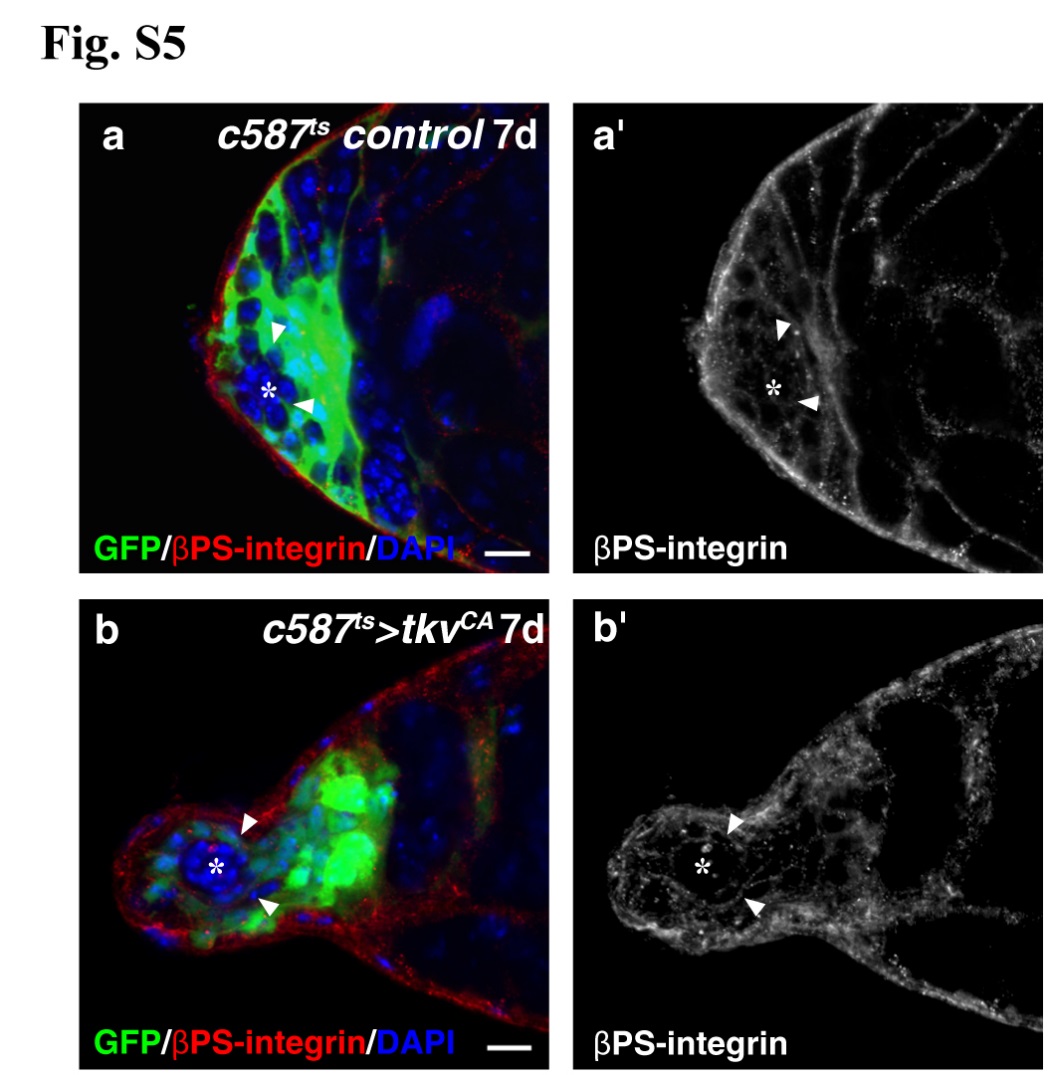


**Supplementary Figure 5.** Induction of *tkv^CA^* does not affect the levels of βPS-integrin. **a** βPS-integrin in *c587^ts^* control testis (red, white arrowheads). **b** βPS-integrin in *c587^ts^>tkv^CA^* testis (white arrowheads). The hub is marked by white asterisk, GFP in green, blue indicates DAPI staining for DNA. Scale bars: 10 μm.


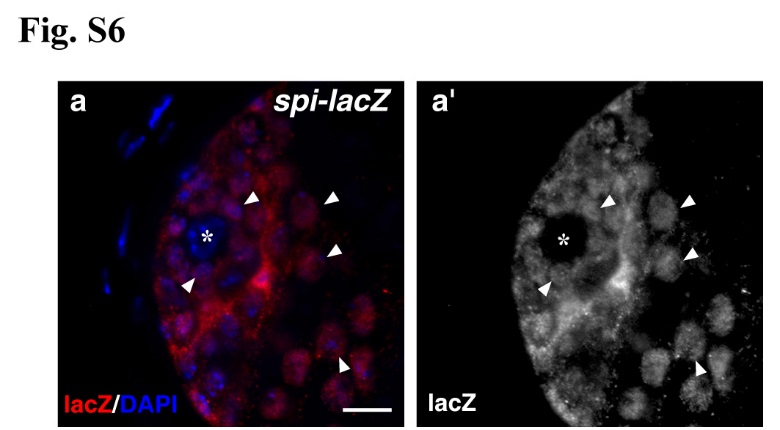


**Supplementary Figure 6.** *spi* is expressed in the germline cells. **a** *spi* (by *spi-lacZ*, red) is expressed in the germline cells (white arrowheads). A separate channel of lacZ staining in black-white (**a'**). The hub is marked by white asterisk, blue indicates DAPI staining for DNA. Scale bar: 10 μm.
